# The Tissue Distribution of Four Major Coumarins after Oral Administration of Angelicae Pubescentis Radix Extract to Rats Using Ultra-High-Performance Liquid Chromatography

**DOI:** 10.1155/2019/2365697

**Published:** 2019-02-07

**Authors:** Yuanyuan Ge, Shujing Chen, Qian Luo, Chun-peng Wang, Jia Hao, Jun He, Xiaopeng Chen, Xuejing Yang, Jin Li, Yan-xu Chang

**Affiliations:** ^1^Tianjin Key Laboratory of Phytochemistry and Pharmaceutical Analysis, Tianjin University of Traditional Chinese Medicine, Tianjin, 300193, China; ^2^Tianjin State Key Laboratory of Modern Chinese Medicine, Tianjin University of Traditional Chinese Medicine, Tianjin, 300193, China; ^3^School of Pharmacy, Harbin University of Commerce, Harbin 150076, China; ^4^Key Laboratory of Formula of Traditional Chinese Medicine (Tianjin University of Traditional Chinese Medicine), Ministry of Education, Tianjin, 300193, China

## Abstract

Angelicae pubescentis radix (APR) is widely applied in treating rheumatoid arthritis in China. Coumarins are the major active compounds of APR extract including columbianetin, columbianetin acetate, osthole, and columbianadin. The* in vivo* behavior of the four major coumarins of APR has not been systematically reported. A feasible and reliable ultra-performance liquid chromatography (UPLC) method was established and validated for the quantification of the above four coumarins in rat various tissues (including heart, liver, spleen, lung, kidney, uterus, ovary, and muscle) after oral administration of APR extract. The separation was implemented on a Waters ACQUITY BEH C18 column (4.6 mm × 100 mm, 1.7 *μ*m) with gradient mobile phase comprising acetonitrile-water (with 1mM formic acid) at a flow rate of 0.3 mL/min. The tissue homogenate samples were prepared by liquid-liquid extraction with ethyl acetate. The calibration curves were linear in the range of 1.6-20000 ng/mL for four coumarins with the lower limit of quantitation of 1.6 ng/mL in rat tissues. The intraday and interday precisions and recoveries were all within 80-100% with the relative standard deviations (RSDs) which were all less than 10.9%. The method was successfully applied to the tissue distribution research after oral administration of 6.0 g/kg APR extract to rat. The results revealed that the tissues distributions of four coumarins were in the liver, followed by the ovary, uterus, kidney, lung, heart, spleen, and muscle.

## 1. Introduction

Traditional Chinese Medicine (TCM) has been crucial to prevent and treat diseases. TCM was applied to treat the illness depending on the theory of Traditional Chinese Medicine, which contains viscera-state theory [1]. According to the cure rules of chemical drugs, the tissues are the functional unit which maintains the homeostasis. In consideration of the function level, the viscera-state theory has similar effect with tissue level restoration in treating diseases. It can be seen that the tissue distribution study might act on the elucidation of healing mechanism of TCM.

Angelicae pubescentis radix (APR) originates in the root of* Angelica pubescens Maxim*.* f. biserrata Shan et Yuan*, which is primitively recorded in “Shennong's Herbal Classic” [2]. APR possesses the efficacy of dispelling wind and removing obstruction in the meridians, activating blood and relaxation tendons, dispersing cold, and relieving pain [3], especially in treating anemofrigid-damp arthralgia. The target tissue distribution of chemical ingredients should be taken into account to find out the pharmacological mechanism of APR.

Phytochemical study has demonstrated that coumarins are the main constituents in APR [4]. Previous study demonstrated that the representative coumarins of APR include the columbianetin, columbianetin acetate, osthole, and columbianadin [4, 5]. Columbianetin was found to possess the characteristic of phytoalexin [6], anti-inflammatory [7, 8], inhibitory lipid peroxidation, and platelet aggregation effects [9]. Columbianetin acetate proved to have anti-inflammatory and analgesic activities [10] and antitumor effect [11]. Osthole has the antitumor effect on ovarian cancer cells [12], estrogen-like activity in MCF-7 cells, and stimulatory effect on ALP activity of Saos-2 osteoblast cells [13], is a dual agonist of peroxisome proliferator-activated receptor (PPAR) *α*/*γ*, and decreases the hepatic lipid accumulation [14]. Columbianadin was certified to show the inhibitory macrophage activation, stabilize the detachment of alveolar macrophages [15], and induce apoptosis and necroptosis in HCT116 colon cancer cells [16]. Therefore, the four representative active coumarins in APR play essential roles in disease treatment. The research of* in vivo* behavior of these coumarins is crucial to the understanding of the pharmacological activity of APR extract.

Although the pharmacokinetics or tissue distributions of osthole, columbianadin, and columbianetin in other plant extracts have been studied in previous literature [17–20], the tissue distribution of holistic representative coumarins in APR has not been reported. In order to explore the relation of viscera-state theory and tissue contribution of APR for further illuminating the mechanism, a sensitive, precise, and reliable UPLC method was established to test simultaneously the concentration of columbianetin, columbianetin acetate, osthole, and columbianadin in different tissues after oral APR extract to rat tissues.

## 2. Experimental

### 2.1. Chemicals and Reagents

Columbianetin, columbianetin acetate, osthole, and columbianadin were obtained from National Institute for the Control of Pharmaceutical and Biological Products (Beijing, China). Acetonitrile (HPLC grade) was purchased from Dikma Technologies Inc. (Dikma, USA). Methanol (HPLC grade) and ethyl acetate (analytical grade) were purchased from Tianjin Concord Science Co. Ltd. (Tianjin, China). Formic acid was purchased from Merck (Darmstadt, Germany). Deionized water was purified with a Milli-Q Academic ultra-pure water system (Millipore, Milford, MA, USA).

### 2.2. Instrument and Analytical Conditions

The liquid chromatography of Waters UPLC (Waters, America) system was used with the photodiode array detection (PDA). Chromatographic separation was performed on an ACQUITY BEH C18 column (4.6 mm × 100 mm, 1.7 *μ*m) with a security VanGuard C18 column (2.1 mm × 12.5 mm, 1.7 *μ*m). The column oven temperature was maintained at 25°C. The mobile phase was composed of acetonitrile (A) and aqueous formic acid (1mM) (B) using gradient elution as follows: 40% A at 0-5 min, 40-70% A at 5-10 min, 70-75% A at 10-11 min, 75-90% A at 11-19 min, 90% A at 19-22 min, and 90-40% A at 22-29 min. The flow rate was 0.3 mL/min and the injection volume was 5 *μ*L. The wavelength was set at 320 nm for the four coumarins.

### 2.3. Preparation of APR Extract

**Figure 1:**
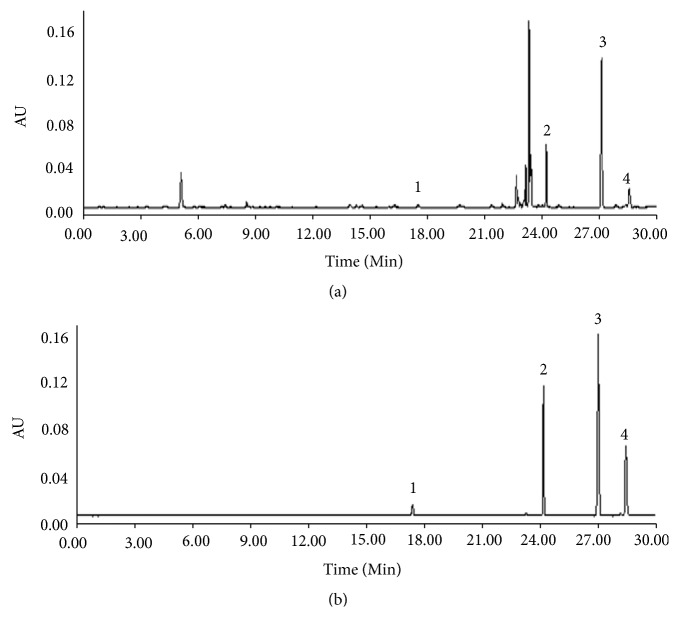
The UHPLC-PDA chromatograms with APR extract sample (a) and standard solution of four compounds (b). 1: columbianetin, 2: columbianetin acetate, 3: osthole, 4: columbianadin.

For the preparation of the extract, 2 kg APR was cut into pieces and soaked with 75% ethanol (16L) for 2 h. The filtrate was concentrated by using a rotary evaporator under reduced pressure. The dry APR extract (100 mg) was accurately weighed in triplicate. The sample was dissolved with 5 mL of 70% methanol by using an ultrasonic bath for 30min and then cooled at room temperature. The supernatant of 2 mg/mL APR extract was injected into the UPLC-PDA system. The chromatography conditions of UPLC-PDA for separation and determination of four coumarins in APR extract were slightly modified according to previous studies [21]. The mobile phase consisted of acetonitrile (A) and 0.1% formic acid/water (B) set as follows: 0–2 min, 5–8% A; 2–5 min, 8–10% A; 5–8 min, 10–14% A; 8–9 min, 14–16% A; 9–12 min, 16–16% A; 12–13 min, 16–18% A; 13–14 min, 18–20% A; 14–15 min, 20–21% A; 15–18 min, 21–23% A; 18–21 min, 23–30% A; 21–23 min, 30–50% A; 23–28 min, 50–51% A; 28–29 min, 51–95% A; and 29-30 min, 95-5% A. The injection volume was 2 *μ*L. The fingerprint of APR extract was shown in Figure 1. The standard concentrations ranges were 0.4–50 *μ*g/mL for columbianetin, 1.6–200 *μ*g/mL for columbianetin acetate, 4.0–500 *μ*g/mL for osthole, and 1.2–150 *μ*g/mL for columbianadin, respectively. The contents of columbianetin, columbianetin acetate, osthole, and columbianadin were 0.4842 mg/g, 3.559 mg/g, 14.63 mg/g, and 1.059 mg/g in APR extract, respectively. The osthole was the most abundant coumarin in APR extract.

### 2.4. Preparation of Stock Solutions and Quality Control Samples

**Figure 2:**
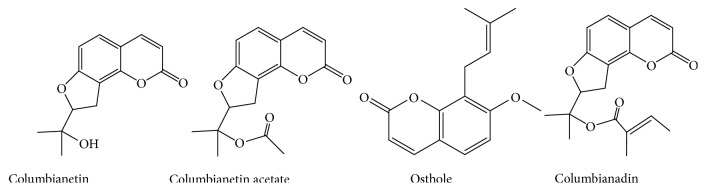
Chemical structures of columbianetin, columbianetin acetate, osthole, and columbianadin.

Four stock solutions of columbianetin, columbianetin acetate, osthole, and columbianadin (1 mg/mL) were prepared in methanol and stored in 4°C, individually. The chemical structures of columbianetin, columbianetin acetate, osthole, and columbianadin are shown in Figure 2. The stock solutions of above compounds were stepwise diluted with methanol, and then the diluents were added to blank tissues to yield final concentrations of 1.6, 8, 40, 200, 500, 2500, 10000, and 20000 ng/mL. Quality control (QC) samples were prepared with blank tissues at the concentrations of 160, 500, and 2000 ng/mL for osthole and columbianadin; 80, 400, and 2000 ng/mL for columbianetin acetate; 80, 500, and 2000 ng/mL for columbianetin, respectively.

### 2.5. Preparation of Tissue Samples

The tissue samples were homogenized with 2-fold volume of 0.9% normal saline. The tissue homogenate was processed by liquid-liquid extraction. An aliquot of 200 *μ*L tissue homogenate was put into 1.5 mL eppendorf tube. The 1000 *μ*L of ethyl acetate was added to extract the four analytes. The tube was vortex-mixed for 1 min and centrifuged at 14000 rpm for 10 min. The supernatant was transferred into another tube and evaporated to dryness under nitrogen at 40°C. The dried residue was reconstituted in 80 *μ*L of methanol. After swirling for 1 min, oscillating ultrasonically for 3 min, and centrifuging at 14000 rpm for 10 min, 5 *μ*L aliquot of the supernatant was injected into UPLC for analysis.

### 2.6. Method Validation

According to the USFDA bioanalytical method validation guidelines [22], the method was validated in terms of specificity, sensitivity, linearity, accuracy, precision, and recovery. The specificity was assessed using blank tissue samples, blank tissue spiked with standard analytes, and real tissue samples after oral administration of APR extract. Lower limit of quantitation (LLOQ) means the concentration of minimum signal to noise ratio was at least more than five for biosamples. Calibration curve samples of four coumarins were handled with blank rat tissue homogenate at the concentrations of 1.6, 8, 40, 200, 500, 2500, 10000, and 20000 ng/mL. The stand curve was gained by plotting the peak area (y) ratio of nominal concentration (x) with least-squares linear regression using 1/x^2^ as weighing factor. The precision and accuracy were calculated by determining QC samples of low, middle, and high levels. The precision was expressed by the relative standard deviations (RSDs), and the accuracy was represented by the calculated concentration ratio of known concentration. The extraction recovery was described by comparing peak areas of analytes from extracted samples with the postextracted spiked samples. The stability of processed samples was estimated by analyzing the target compounds which were kept in an autosampler for 24 h.

### 2.7. Application to Tissue Distribution Study

Female Sprague-Dawley rats (250~280g) were fed under standard housing conditions and were acclimated at breeding room for at least one week beforehand. The rats were fasted for 12h before the experiment. All rats experiments were realized in strict accordance with the Guidelines for the Care and Use of Laboratory Animals and were approved by the Animal Ethics Committee of Tianjin University of Traditional Chinese Medicine (Tianjin, China). The 18 rats were divided randomly into three groups (n=6 per group). The rats were sacrificed by cervical dislocation at 4, 6, and 8 h after oral administration of 6 g/kg APR extract, respectively. The heart, liver, spleen, lung, kidney, uterus, ovary, and muscle were rapidly excised, rinsed, wiped, and weighed. All tissues were stored at -80°C until analysis.

## 3. Results

### 3.1. Method Validation

#### 3.1.1. Specificity and Selectivity

**Figure 3:**
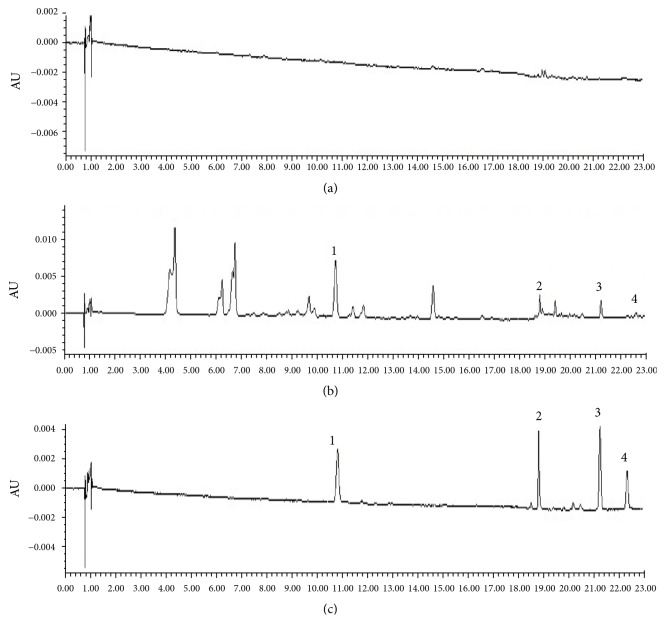
Representative tissue chromatogram of (a) blank heart tissue, (b) blank heart tissue spiked with the standard compounds at LLOQ, and (c) real samples after administration of APR extract. 1: columbianetin, 2: columbianetin acetate, 3: osthole, 4: columbianadin.

The representative chromatograms consist of blank tissues homogenate, blank biosample with standard substances, and tissues samples after oral administration of APR extract as shown in Figure 3. The results revealed that the retention time of columbianetin, columbianetin acetate, osthole, and columbianadin was 10.9, 19.0, 21.3, and 22.4 min, respectively. As shown in the chromatograms, there are no obvious endogenous substances and metabolite to disturb the quantification of the four coumarins.

#### 3.1.2. Linearity and LLOQ

The calibration curves were over the range of 1.6-20000 ng/mL, and the correlation coefficients (r) were more than 0.99. These indicated that calibration curves exhibited good linearity to quantify the four coumarins. The lower limit of quantitation (LLOQ) of four analytes was set at 1.6 ng/mL with the S/N value approximating 5.

#### 3.1.3. Precision and Accuracy

The results of the precision and accuracy of the four analytes are summarized in Table 1. The intraday precisions in eight tissues ranged from 0.35% to 5.77%, and corresponding accuracies ranged from 89.2% to 97.3%. The interday precisions ranged from 0.85% to 8.71%, and corresponding accuracies ranged from 90.4% to 98.3%. The data revealed that the method was precise and accurate for quantitation of four coumarins in tissue samples.

#### 3.1.4. Recovery

The results of recovery are listed in Table 2. The extraction recoveries of analytes in eight tissues were within the range of 80.2%-99.9%, and corresponding RSDs are less than 15%. The data indicated that the interference could be neglected in rat tissue sample.

#### 3.1.5. Stability

The stability of processed samples which were kept in an autosampler for 24 h is shown in Table 1. The accuracy ranged from 88.4% to 99.8%, and corresponding RSD ranged from 2.55% to 12.8%. The data were well within the acceptable limit. The results demonstrated that the four compounds were stable under the detected conditions.

### 3.2. Tissue Distribution Studies

**Figure 4:**
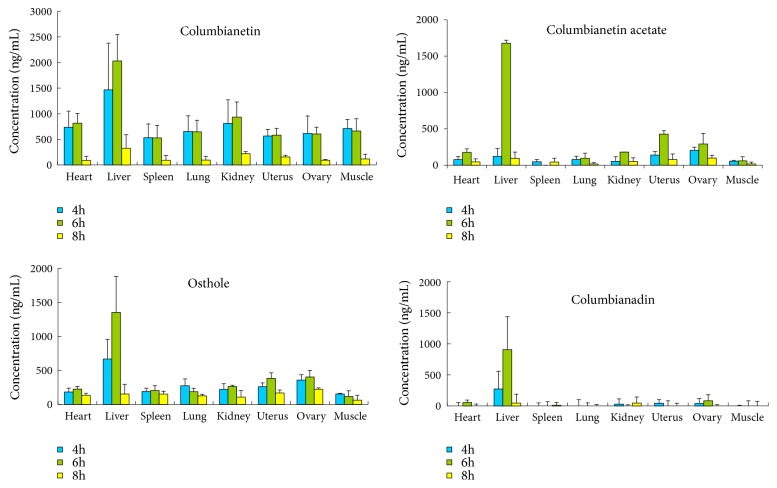
The tendency graph of columbianetin, columbianetin acetate, osthole, and columbianadin in eight tissues at 4, 6, and 8 h after oral administration of APR extract (n=6).

The tissue distributions of the four coumarins were investigated at 4, 6, and 8 h after given orally 6.0 g/kg APR extract to female rats. The examined tissues included the heart, liver, spleen, lung, kidney, uterus, ovary, and muscle. The concentrations of four coumarins in tissues are presented in Table 3. The mean concentration of four analytes at different tissues and points-in-time are shown in Figure 4. The highest and lowest concentrations of four coumarins were observed at 6 h and at 8 h in different tissues from 4 h to 8 h, respectively. The phenomenon indicated that the distribution tendency of four coumarins rose first then fell in most tissues. The reason that the highest concentration of four coumarins was detected in the liver at 6 h after oral administration APR extract was related to the liver in which the drugs metabolism mainly takes place. The concentration of columbianetin was higher than those of other constituents in muscle, which implied that columbianetin might be the main activity constituent in APR to cure arthrophlogosis. The relative higher distribution in kidney suggested that it might be the primary excretion tissue of the columbianetin. On the whole, the concentration of columbianetin was higher than other compounds, and the concentration order of four coumarins was columbianetin>osthole>columbianetin acetate > columbianadin in eight tissues, successively. The concentration of columbianetin acetate and osthole in uterus and ovary was higher than those in other tissues, which probably implied that both components exert estrogen-like effect in uterus and ovary. This result deeply proved the effect of estrogen-like activity of osthole in MCF-7 cells.

## 4. Conclusion

A sensitive, reliable, and fast method was established and validated for the simultaneous tissue distribution of osthole, columbianetin, columbianetin acetate, and columbianadin after oral administration of APR extract in rat. The tissue distribution provides a process to understand the pharmacological activity of APR* in vivo*. The results showed that the four coumarins were mainly distributed in liver which is the drugs metabolism place. The main distributed ingredients are columbianetin and osthole; next are columbianetin acetate and columbianadin in various tissues. The results potentially imply that columbianetin and osthole are the active constituents in APR for curing rheumatoid arthritis. The tissues distribution of columbianetin, columbianetin acetate, osthole, and columbianadin would be meaningful for study of Angelicae pubescentis radix on the clinical application.

## Figures and Tables

**Table 1:** The precision and stabilities of columbianetin acetate, columbianetin, osthole, and columbianadin.

	Concentration (ng/mL)	Intra-day precision		Inter-day precision		Autosampler for 24 hours stability	
		Accuracy(%)	RSD(%)	Accuracy(%)	RSD(%)	Accuracy(%)	RSD(%)
Columbianetin	80	95.9	5.20	94.2	8.71	99.8	7.83
	500	93.9	0.35	96.2	2.11	97.9	2.89
	2000	93.8	0.74	97.3	1.91	96.0	9.48

Columbianetin acetate	80	93.6	3.05	93.5	7.32	97.4	2.55
	400	92.6	3.04	95.6	0.85	96.7	7.63
	2000	91.8	5.71	90.4	1.89	85.4	3.14

Osthole	160	97.3	5.29	98.3	4.32	99.2	5.28
	500	89.2	0.91	91.6	1.67	97.1	12.8
	2000	91.9	2.68	93.6	2.64	88.4	5.39

Columbianadin	160	92.6	5.77	93.8	6.54	97.1	5.73
	500	93.4	0.51	95.8	2.32	99.1	3.30
	2000	93.7	0.74	96.9	1.47	95.6	8.74

**Table 2:** The recoveries of columbianetin, columbianetin acetate, osthole, and columbianadin.

Tissues	columbianetin						columbianetin acetate					
	Low		Middle		High		Low		Middle		High	
	Recovery	RSD	Recovery	RSD	Recovery	RSD	Recovery	RSD	Recovery	RSD	Recovery	RSD
	(%)	(%)	(%)	(%)	(%)	(%)	(%)	(%)	(%)	(%)	(%)	(%)

Heart	81.8	3.96	94.6	1.72	93.8	0.69	92.8	3.80	82.8	4.74	83.8	2.55
Liver	87.8	2.67	88.7	0.72	94.2	1.57	85.7	7.03	82.1	10.5	87.1	2.97
Spleen	89.5	4.09	99.1	1.55	97.4	1.93	80.8	9.63	80.7	3.92	93.2	1.19
Lung	97.7	2.58	99.4	8.71	97.8	1.46	84.3	10.3	85.8	9.46	86.1	5.15
Kidney	97.4	4.64	95.8	5.80	95.5	0.61	84.6	4.71	81.1	4.75	91.7	3.02
Uterus	93.0	8.54	95.7	8.83	97.3	3.65	97.6	1.02	86.2	4.13	88.4	7.43
Ovary	99.3	5.82	92.6	3.28	97.9	7.40	83.1	10.4	85.7	5.75	89.5	5.21
Muscle	92.1	6.42	93.9	0.35	93.8	0.74	91.3	6.04	98.3	3.22	90.1	5.71

Tissues	osthole						columbianadin					
	Low		Middle		High		Low		Middle		High	
	Recovery	RSD	Recovery	RSD	Recovery	RSD	Recovery	RSD	Recovery	RSD	Recovery	RSD
	(%)	(%)	(%)	(%)	(%)	(%)	(%)	(%)	(%)	(%)	(%)	(%)

Heart	91.2	7.70	98.6	2.75	94.3	3.55	82.1	1.83	80.2	4.73	88.4	1.13
Liver	93.1	4.42	94.6	5.87	93.1	5.93	81.3	4.84	81.9	1.92	84.3	5.68
Spleen	95.1	6.40	95.7	7.61	90.9	3.33	81.0	3.11	83.1	6.19	91.9	2.00
Lung	94.3	9.37	99.8	1.38	92.3	5.74	96.5	7.32	89.0	1.32	90.0	3.05
Kidney	94.9	10.88	97.1	4.08	95.5	7.42	92.2	1.73	95.3	1.26	91.4	0.81
Uterus	88.2	10.51	96.5	2.33	99.9	5.11	94.3	9.57	99.2	2.72	96.5	7.75
Ovary	93.8	3.76	94.7	6.11	93.9	5.63	96.8	3.41	98.4	4.09	98.4	7.47
Muscle	97.3	5.29	89.2	0.90	91.9	2.68	92.6	5.77	98.2	4.76	97.6	3.20

**Table 3:** The concentrations of columbianetin, columbianetin acetate, osthole, and columbianadin in tissues (ng/mL)(n=6).

Tissues	columbianetin			columbianetin acetate			osthole			columbianadin		
	4h	6h	8h	4h	6h	8h	4h	6h	8h	4h	6h	8h
Heart	735.0±316.5	814.6±192.3	86.9±80.2	77.4±41.2	175.0±50.0	45.3±40.7	183.5±56.0	227.4±39.3	135.5±28.6	0.00±0.00	54.3±36.8	0.0±0.0
Liver	1467.0±913.4	2032±514	325.4±262.5	183.3±22.2	1677.0±42.4	94.0±86.4	669.3±286.5	1352±529.0	257.8±24.3	273.2±185.4	908.4±738.6	46.8±51.3
Spleen	533.1±266.9	529.1±243.6	171.0±9.9	47.4±30.6	180.9±0.0	42.8±53.4	191.2±48.5	206.6±67.6	153.5±42.6	0.00±0.00	0.00±0.00	12.2±24.4
Lung	653.0±306.1	647.4±227.5	95.3±72.0	76.6±46.9	94.5±70.9	16.9±17.7	275.0±101.6	188.3±49.0	125.8±24.7	0.00±0.00	0.00±0.00	0.0±0.0
Kidney	810.3±460.5	933.7±295.8	222.1±41.5	104.7±45.7	180.9±0.0	50.5±40.9	221.2±86.1	264.1±19.6	162.5±38.2	136.3±60.9	0.00±0.00	142.2±47.4
Uterus	565.1±129.8	582.6±130.5	155.1±34.8	140.5±49.2	428.3±47.0	77.7±76.9	261.0±57.0	382.6±83.5	167.0±42.3	133.7±44.6	0.00±0.00	0.00±0.00
Ovary	614.6±341.2	604.9±132.2	91.4±16.3	204.8±42.6	292.9±145.0	98.6±38.8	358.5±78.3	403.5±97.6	224.0±20.2	118.7±68.5	126.0±9.7	0.00±0.00
Muscle	712.2±176.4	663.3±239.3	117.4 ±92.8	55.0±10.7	79.7±49.4	32.9±31.7	154.7±9.7	118.1±82.9	126.4±7.0	0.00±0.00	0.0±0.0	0.00±0.00

## Data Availability

The data used to support the findings of this study are included within the article.
